# Case of Rupture of the Uterus

**Published:** 1854-11

**Authors:** J. S. Monell

**Affiliations:** New York


Case of Rupture of the Uterus.—*Recovery.*—By J. S. Monell, M. D., of New York. I was called on Tuesday, July 15th, about 2 o'clock P. M., to see a patient, aged thirty-five years, in labor with her fifth child, who had been, since 1 o'clock of the night previous, under the care of a midwife. The membranes had ruptured about an hour before my visit; and upon examination, a hand was found protruding from the vulva, and above this, the fœtus presenting by the face. The pains were excessively severe.

Circumstances, over which I had no control, delayed the adoption of the only means of relief, until between 4 and 5 o'clock. By this time the pains had ceased entirely, and the aspect of the patient was alarming; the countenance was palid, the lips blue, pulse quick and feeble, with abdominal tenderness. Through the abdominal parietes, the contour of the child was readily distintinguishable, especially in the right hypochondrium. Upon vaginal examination, the head was found to have receded.

At this time the patient was seen by several medical gentlemen, who all agreed as to the correctness of the diagnosis of the case as one of ruptured uterus; and immediate delivery was resolved on as furnishing the only feasible hope of saving life.

Version was effected without difficulty, by Dr. T. F. Cook, who found the laceration in the right side of the uterus, the foot having passed through the rent into the peritoneal cavity. At the time of delivery, the case appeared nearly hopeless; the countenance was pale and haggard, the pulse rapid and thready; frequent vomiting, and tenderness over the abdomen, indicated the probable existence of peritonitis.

Nevertheless, soon after delivery, and by the active administration of stimulants, the patient began to rally, and in about two hours, the use of Majendie's solution of morphine in fifteen drop doses, was commenced, to be continued every two hours until sleep should ensue.

The patient slept well during the night, and was found on Wednesday morning, with a pale and anxious countenance, tongue furred white, pulse 100, hard and quick, skin hot and dry, tenderness and tumefaction of the abdomen. She had passed no urine, and the lochia were suppressed. Motion, and even respiration produced abdominal distress. She was directed to take every three hours, a tablespoonful of a mixture of spts. minderer., spts. nitr., ipecac., and Majendie's solution, with hot fomentations to the abdomen.

In the evening, no special mitigation of symptoms was apparent; but the urine was voided after applications of flannel cloths wrung out in hot water, had been freely used. To take Dover's powder, gr. xij., ext. hyoscy. grs. i., calomel, grs. ij., at bed time, and to continue fomentations to the abdomen.

Thursday, 17th. No improvement. Lochia but small in amount, pulse 104, incompressible, countenance pale and anxious, respiration sighing, tongue heavily coated with a thick, white fur; patient complains of feeling faint, and also of great tenderness over the seat of laceration. Stimulants were given internally, and two leeches applied over the seat of pain, which produced marked beneficial effect. Fomentations of hops and chamomile flowers were used; and the lochial discharge became more profuse, though quite fœtid. In the evening, the pulse was reduced to 90, the countenance more natural, and the tenderness over the uterus was diminished. She had slept three hours, and was much refreshed. Calomel, gr. ij., with pulv. Doveri, gr. x., to be taken at bed-time.

Friday, 18th. Much improved. Had slept well. The pulse was 88, the skin more natural, and less abdominal tenderness. The fomentations were discontinued, and some light nourishment allowed, the patient having taken for three days cold water or ice only. Anodyne at night, as yesterday.

Saturday, 19th. No special change. Calomel, grs. x. with pulv. Doveri, grs. ix., at night, with the intention of following with a cathartic in the morning, to relieve the bowels, which had not been moved since the day of confinement.

Sunday, 20th. The patient passed a restless night. The abdominal tenderness had increased, and the intestines appeared to be loaded with fecal matter. To relieve this, pulv. purgans was given in half-drachm doses every three hours, until free catharsis was produced, by which she was much relieved.

Convalescence seemed now to be established, and she progressed steadily, though slowly. From this time, to August 1st, she was kept under moderate medication by laxatives and diuretics, with careful attention to the lochia and the bandage. At this time she began to sit up, and though strictly enjoined to avoid exertion, she could not be restrained after August 15th, from her usual duties. Since that time, I have visited her occasionally; and at the present time (Sept. 18th), she declares herself as well as ever. —*N. Y. Med. Times.*

